# Single-cell chromatin accessibility profiling of cell-state-specific gene regulatory programs during mouse organogenesis

**DOI:** 10.3389/fnins.2023.1170355

**Published:** 2023-06-27

**Authors:** Qiuting Deng, Shengpeng Wang, Zijie Huang, Qing Lan, Guangyao Lai, Jiangshan Xu, Yue Yuan, Chang Liu, Xiumei Lin, Weimin Feng, Wen Ma, Mengnan Cheng, Shijie Hao, Shanshan Duan, Huiwen Zheng, Xiaoyan Chen, Yong Hou, Yingjie Luo, Longqi Liu, Chuanyu Liu

**Affiliations:** ^1^College of Life Sciences, University of Chinese Academy of Sciences, Beijing, China; ^2^BGI-Hangzhou, Hangzhou, China; ^3^BGI-Shenzhen, Shenzhen, China; ^4^Shenzhen Bay Laboratory, Shenzhen, China

**Keywords:** mouse organogenesis, single-cell ATAC-seq, spinal cord, paraxial mesoderm, *cis*-regulatory elements

## Abstract

In mammals, early organogenesis begins soon after gastrulation, accompanied by specification of various type of progenitor/precusor cells. In order to reveal dynamic chromatin landscape of precursor cells and decipher the underlying molecular mechanism driving early mouse organogenesis, we performed single-cell ATAC-seq of E8.5-E10.5 mouse embryos. We profiled a total of 101,599 single cells and identified 41 specific cell types at these stages. Besides, by performing integrated analysis of scATAC-seq and public scRNA-seq data, we identified the critical *cis*-regulatory elements and key transcription factors which drving development of spinal cord and somitogenesis. Furthermore, we intersected accessible peaks with human diseases/traits-related loci and found potential clinical associated single nucleotide variants (SNPs). Overall, our work provides a fundamental source for understanding cell fate determination and revealing the underlying mechanism during postimplantation embryonic development, and expand our knowledge of pathology for human developmental malformations.

## Introduction

In mammals, early organogenesis is completed within a short time frame, and cells from the three germ layers can form precursor cells of various organs, which is a fundamental biological question (Downs and Davies, 1993; Waddington, 2012; Kojima et al., 2014). As the precursor cells for all major organ systems, the underlying mechanism of cell proliferation and cell type diversification is still largely unclear. Cell fate decision and cell state transition events during organogenesis, especially neurogenesis and somitogenesis, which are closely related to ubiquitous profiles of epigenetic and transcriptional alteration (Shahbazi and Zernicka-Goetz, 2018; Sagar and Grün, 2020; Shahbazi, 2020).

High-resolution charting of mammalian embryonic development is gradually deepening with the emergence of single-cell sequencing technologies (Dong et al., 2018; Argelaguet et al., 2019; Cao et al., 2019; Nowotschin et al., 2019; Peng et al., 2019; Pijuan-Sala et al., 2019, 2020; Argelaguet et al., 2022; Magaletta et al., 2022; Qiu et al., 2022). These technologies have already been adopted on different development research field, such as the construction of the mammalian embryonic cell interaction atlas (Cao et al., 2019; Pijuan-Sala et al., 2019, 2020) and the studies of germ layer and organ development (Nowotschin et al., 2019; Han et al., 2020; Kelly et al., 2020; Magaletta et al., 2022; Jiang et al., 2023). Nonetheless, most of these studies mentioned above focused on transcriptome analysis and neglected the characterization of chromatin accessibility during organogenesis. Recently, single-cell chromatin accessibility maps have been captured, referring one embryonic stage (Pijuan-Sala et al., 2020). Concerning the early organogenesis period from E8.5 to E10.5, when cell types diversify, the regulatory programs that define organ cell repertoire remain to be elucidated, especially neurogenesis and somitogenesis. Although single-cell transcriptome sequencing has revealed potential mechanisms of neurogenesis and somitogenesis (Farrell et al., 2018; Ibarra-Soria et al., 2018; Christoph et al., 2022), the epigenetic regulatory mechanism of the development from spinal cord and paraxial mesoderm to the dorsal-ventral axis and skeleton remains unclear.

Here, we applied a high-throughput single-cell sequencing assay for transposase-accessible chromatin (scATAC-seq) to generate a chromatin accessibility dataset and performed an integrated multiomics analysis of chromatin accessibility and gene expression during early organogenesis. As a result, we identified 101,599 single cells and 41 cell types, which could be a valuable resource for revealing embryonic developmental mechanisms, preventing miscarriage, and improving pregnancy.

## Methods

### Animal study

All mice experiments were approved by the Institutional Review Board on the Ethics Committee of BGI (Permit No. BGI-IRB A23010). Wild-type C57BL/6 mice (Guangdong medical laboratory animal center) were interbred, noon at the day of a vaginal plug was considered as E0.5. Pregnant females were sacrificed, and 8 E8.5 embryos, 15 E9.5 embryos and 8 E10.5 embryos were collected for scATAC-seq experiments. Mouse embryos were dissected and pre-chilled in cold PBS, followed by cell dissociation and nuclei extraction steps. Total 31 mouse embryos were processed and analyzed in following experiments.

### Cell dissociation and nuclei isolation

To generate a single cell suspension, embryo samples were first digested by 0.2 mg/mL Liberase^™^ (Roche, 5401119001) and 0.025% Trypin (Thermo, 25200056), which were incubated at 37°C for 60 min on a thermo shaker. Dissociated cells were filtered through a 70 μm cell strainer (Falcon, 352350) and a 40 μm cell strainer (Falcon, 352340). Cell suspension was centrifuged for 5 min at 500 g and cells were washed with 0.04% BSA/PBS for 1 or 2 times. Single-nucleus preparations were derived from the Omni-ATAC protocols as previously described (Corces et al., 2017), with some adjustments. In brief, cells were resuspended in 100 μL of chilled cell lysis buffer (CLB; 10 mM Tris–HCl pH7.5, 10 mM NaCl, 3 mM MgCl_2_, 0.1% Tween-20 (Sigma, P9416), 0.1% NP40 (Roche, 11332473001), 0.01% digitonin (Sigma, D141), 1% BSA/PBS), and incubated on ice for 5 min. Subsequently, 1 mL of chilled ATAC resuspension buffer (RSB; 10 mM Tris-HCl pH7.5, 10 mM NaCl, 3 mM MgCl_2_, 0.1% Tween-20, 1% BSA/PBS) was added into the lysed cell suspension, and nuclei were spun down at 500 *g*, 4°C for 5 min. Nuclei was resuspended in 50 μL of BSA/PBS and counted by DAPI staining.

### scATAC-seq library construction and sequencing

scATAC-seq libraries were prepared using DNBelab C Series Single-Cell ATAC Library Prep Set (MGI, 1000021878; Yu et al., 2021). In brief, 100,000 nuclei were resuspended in tagmentation mix which were incubated at 37°C for 30 min with shaking (500 rpm). Then, 10,000 nuclei were input for droplet generation and labeling, followed by droplet pre-amplification and droplet breaking. The magnetic beads carrying the ATAC fragments were subjected to amplification and purification. The indexed sequencing libraries were constructed according to the manufacturer’s guide, and quantified with the Qubit ssDNA HS Assay Kit 3.0 (Invitrogen, Q32854). In total, we constructed 9 libraries from E8.5 embryonic cells, and 10 libraries from E9.5/E10.5 embryonic cells, respectively. All libraries were sequenced using the DIPSEQ T1/T7 platform at China National GeneBank (CNGB) (Huang et al., 2017), scATAC-seq libraries comprise DNA insert with standard paired-end constructs.

### Data pre-processing and quality control

The raw data were processed by PISA^1^. Briefly, the raw reads were aligned to the mouse reference genome (version: mm10) by BWA-MEM (v0.7.15) with default parameters. Reads with mapping quality less than 10 were removed and the PCR duplicates were also removed for each cell of library by Picard (v1.84).^2^ Next, the fragments file for each library was used for the downstream analysis by ArchR (v1.0.1) (Granja et al., 2021). Cells with the TSS enrichment score less than 4 or the number of captured fragments less than 2,000 were removed, and the doublet score was calculated and filtered using the “addDoubletScores” and “filterDoublets” function with parament “filterRatio = 2” by ArchR.

### Clustering of scATAC-seq data

We used the peak matrixes and fragment files to create chromatin assay by “CreateChromatinAssay” function with default parameters in Signac (v1.8.0) (Stuart et al., 2021). Seurat object was created by “CreateSeuratObject” function for each library. Subsequently, all library datasets were merged together by “merge” function. Then, the data were normalized with the Term Frequency-Inverse Document Frequency methods by “RunTFIDF” function. Top variable features were calculated by the “FindTopFeatures” function with “min.cutoff = ‘q70’” and then the singular value decomposition was computed by “RunSVD” function. Next, Uniform Manifold Approximation and Projection analysis was performed by “RunUMAP” function with parameter “dims = 2:30, reduction = ‘lsi’, n.neighbors = 50, min.dist = 0.4.” “FindNeighbors” and “FindClusters” functions with parameters “algorithm = 3, resolution = 3” were performed to produce cell-type clusters. Finally, the gene activity scores for each gene in cells were calculated by “addGeneScoreMatrix” function, and the cluster-specific genes based on gene scores were calculated by “getMarkerFeatures” function with “useMatrix = ‘GeneScoreMatrix’” (FDR ≤ 0.01 and log2FC ≥ 1) in ArchR. Peak calling for each cell type was performed using “addReproduciblePeakSet” function in MACS2. The cell-type specific peaks were calculated by “getMarkerFeatures” function with “useMatrix = ‘PeakMatrix’” (FDR ≤ 0.01 and log2FC ≥ 1). Motif enrichment analysis was performed by “peakAnnoEnrichment” function with “cutOff = ‘FDR ≤ 0.01 & Log2FC ≥ 0.5.’”

### Integration of scATAC-seq and scRNA-seq data at corresponding stages

We performed integrative analysis of our scATAC-seq dataset with previously published scRNA-seq data of entire mouse embryos from three developmental stages accordingly. First, the expression matrix of scRNA-seq data (GEO accession number: GSE186068 and GSE186069) (Qiu et al., 2022) was downloaded and the cell number of the dataset was downsampled to 1,500 for each cell type of three developmental stages. Then, for each developmental stage, we performed label transferred from the well annotated cell types in scRNA-seq data to our gene activity score matrix by “FindTransferAnchors” and “TransferData” function in Seurat (v4.2.0) (Hao et al., 2021). Besides, the scRNA-seq data was also integrated with the scATAC-seq data by “addGeneIntegrationMatrix” function in ArchR (Granja et al., 2021). Last, peak-to-gene linkage was identified by the “addPeak2GeneLinks” function with “reducedDims = ‘IterativeLSI.’”

### Integration of scATAC-seq and stereo-seq data at corresponding stages

We performed integrative analysis by Tangram package (Biancalani et al., 2021) to integrate our scATAC-seq dataset with previously published spatial transcriptome data generated by Stereo-seq in E9.5 and E10.5 mouse embryos (Chen et al., 2022). Briefly, processed datasets of two sections (E1S2 and E2S1) were downloaded from https://db.cngb.org/stomics/mosta/. Then the maker genes list for each cell type of the mouse embryo from a previous study (Qiu et al., 2022) were collected and used as training genes for Tangram. Next, we mapped the scATAC-seq gene scores data onto spatial gene expression data by the “map_cells_to_space” function in Tangram. Additionally, the corresponding stages of scRNA-seq data were also mapped onto Stereo-seq data with the same strategy.

### Trajectory analysis

We performed the trajectory analysis using “addTrajectory” function with the given cell-types order in ArchR. In addition, “getTrajectory” and “plotTrajectoryHeatmap” functions were used to perform pseudo-time heatmaps for gene scores, gene expression, and motifs.

### Gene ontology analysis

Gene ontology (GO) analysis was performed by “enrichGO” function in clusterProfiler (v3.12.0) (Wu et al., 2021), and *p*-value < 0.05 was used to identify significantly enriched GO terms. In addition, GREAT (v.4.0.4) (Mclean et al., 2010) gene ontology was used to enrich the gene ontology terms for cell types specific peaks.

### LDSC

To calculate heritability and genetic correlations across human diseases/traits in differentially accessible peaks for each cell type from mouse embryonic stages, we used the LDSC package (Bulik-Sullivan et al., 2015) which calculates the enrichment of heritability in a set of annotated SNPs, while considering a baseline model that accounts for the non-random distribution of heritability across the genome. Firstly, we used liftOver tool (v1.2.0) to convert mouse marker peaks of each cell type to the human orthologous genome coordinates (hg19). The detailed commands are as follows: “liftOver input.mouse.celltype.markerPeaks.bed mm10ToHg19.over.chain.gz output.mm10tohg19.orthologous.bed output.unlifted.bed-minMatch = 0.1.” Secondly, we performed LDSC analysis to link human SNPs from GWAS data to orthologous coordinates in the mouse. The the LD scores were calculated by the “make_annot.py” and “ldsc.py” functions according to the tutorial.^3^ Then, the coefficient *p* values (coefficient *p* < 0.01) were used to evaluate the association of the traits/diseases with each cell type. Next, we converted the human SNPs associated with each cell type’s traits/diseases to mouse genome coordinates by liftOver. Then we intersected the SNP loci with the mouse marker peaks regions. This enabled us to identify the orthologous GWAS loci within the marker peaks of each cell type.

## Results

### Characterizing cell types of early mouse organogenesis

**Figure 1:**
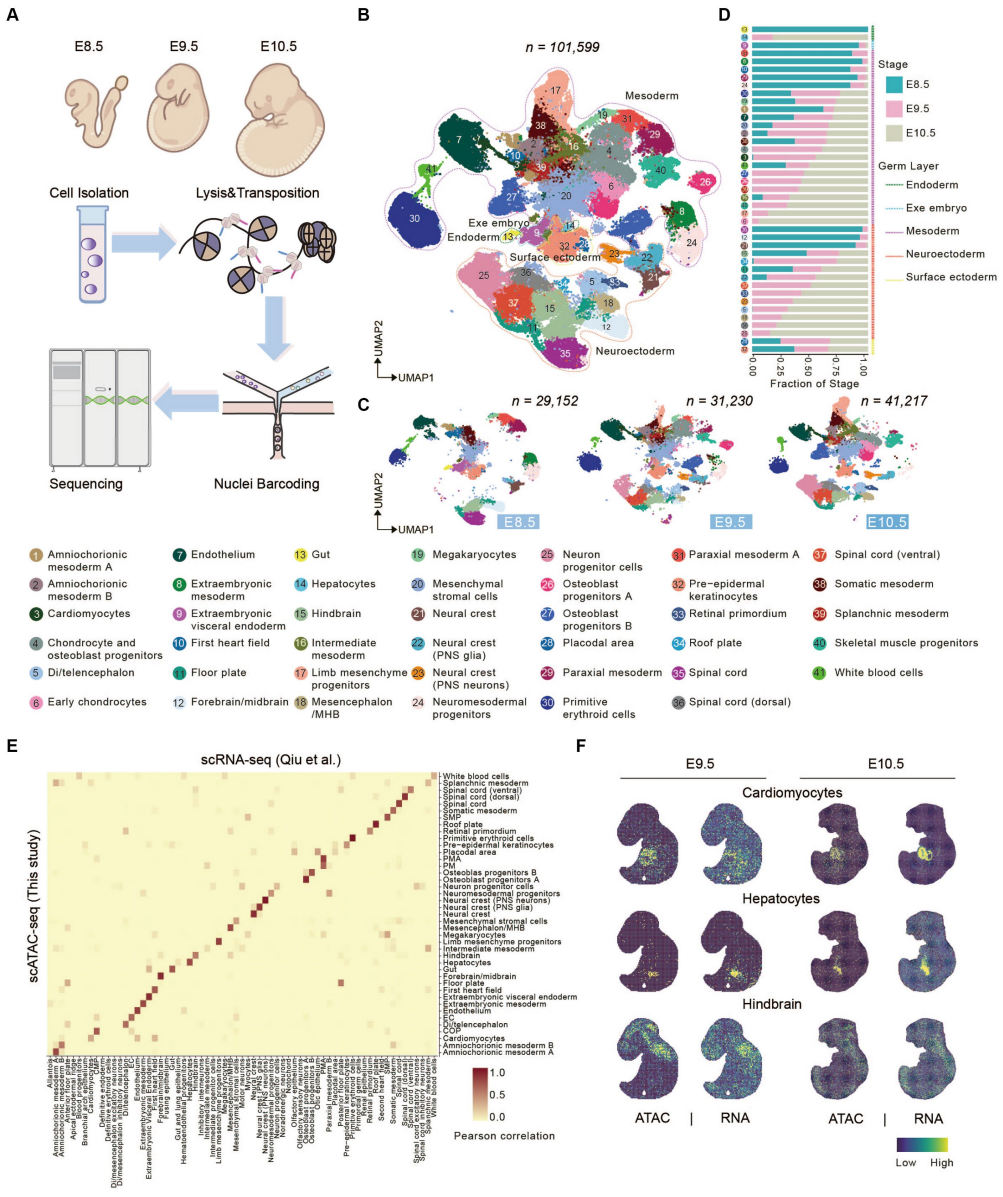
Characterizing canonical cell types in early mouse organogenesis. **(A)** Schematic overview of the approach for generating scATAC-seq data. Mouse embryo from E8.5-E10.5 was dissociated for scATAC-seq. **(B,C)** UMAP visualization of merged **(B)** and embryonic stage **(C)** mouse embryo cells from E8.5-E10.5 (cell number from left to right: *n* = 29,152 for E8.5; 31,230 for E9.5; 41,217 for E10.5). Dots indicate individual cells; cells are colored by cell types. Dashed lines show the distribution of the germ layers. **(D)** Bar plots show the fraction of stages across each cell type. **(E)** Heatmap showing the correspondence between cells annotated by snATAC-seq and cells predicted by scRNA-seq cell-type. **(F)** Tangram inferred spatial distributions of specific cell types from scATAC-seq and scRNA-seq data (Qiu et al., 2022).

To address how chromatin states shape the developing spinal cord and somitogenesis, we performed scATAC-seq of mouse embryonic cells from E8.5 to E10.5 by DNBelab C4 ATAC-seq platform (Figure 1A; Yu et al., 2021). After passing through a stringent quality control pipeline, we obtained a dataset which contained a total of 101,599 cells with a median of 14,291 unique fragments per cell for further analysis (see Methods and Supplementary Table S1). To further evaluate data quality of our dataset, we calculated fraction of reads in peak regions (FRiP) and Transcription Start Site (TSS) enrichment score of each single-cells. A median FRiP of 62.75% in total 244,314 peaks and a median TSS enrichment score of 14.66% were obtained in assayed cells (Supplementary Figure S1), indicating good data quality.

Next, we used Signac R (Stuart et al., 2021) to perform unsupervised clustering of scATAC-seq dataset and identified a total of 69 clusters (Supplementary Table S1). To interpret gene expression level through chromatin accessibility profile, gene activity scores were calculated by summing the number of fragments in each gene’s promoter and gene body regions (Granja et al., 2021). Based on the gene activity score of some marker genes, we clustered 41 cell types, which included cells from all three germ layers (endoderm, mesoderm and ectoderm) and extraembryonic region (Figure 1B and Supplementary Figure S2). Cells from three developmental stages were well integrated without obvious batch effect, and its’ portion in each cluster change coordinately (Figures 1C,D). For example, gut, visceral endoderm, first heart field, spinal cord, and so on accounted for a relatively large proportion in E8.5 and gradually reduced or disappeared at E9.5. On the other hand, neuron progenitor cells, hepatocytes, chondrocyte and osteoblast progenitors (COP), cardiomyocytes, limb mesenchyme progenitors, and so on initially appear at E9.5 and greatly expand at E10.5 (Figures 1C,D).

Besides, to benchmark our scATAC-seq technology, we performed integrated analysis comparing our data with previously published datasets. Using scRNA-seq data (Qiu et al., 2022) from corresponding embryonic stages as a reference dataset, we performed label transfer in Seurat. The result shows that cell types defined by scATAC-seq are consistent with those defined by scRNA-seq (Figure 1E), indicating gene activity score positively correlates with gene expression level. We next sough to explore spatial distribution of defined cell types in mouse embryos. Several advanced technologies have been developed recently which enables profiling transcriptomic information in spatial resolution (Dries et al., 2021; Chen et al., 2022). Above all other techniques, Stereo-seq can generate topographic transcriptomic atlas of a whole mid-or late-gestation embryo with high resolution and sensitivity (Chen et al., 2022). We performed integrated analysis using our scATAC data with public MOSTA dataset (mouse organogenesis spatiotemporal transcriptomic atlas) (https://db.cngb.org/stomics/mosta/) generated by Stereo-seq, and mapped our scATAC-seq data onto MOSTA spatial maps (see Section Methods). Data integration showed that the spatial distribution of cell population defined by our ATAC data was consistent with position of corresponding organ, such as cardiomyocytes, hepatocytes, and hindbrain (Figure 1F and Supplementary Figure S3).

Taken together, these results support that we portrayed an informative chromatin landscape for early stage of mouse organogenesis, which enabled interpretation of regulatory roles between chromatin accessibility and gene expression during mouse embryonic development.

### Characterization of cell type-specific regulatory profiles in spinal cord lineages

**Figure 2:**
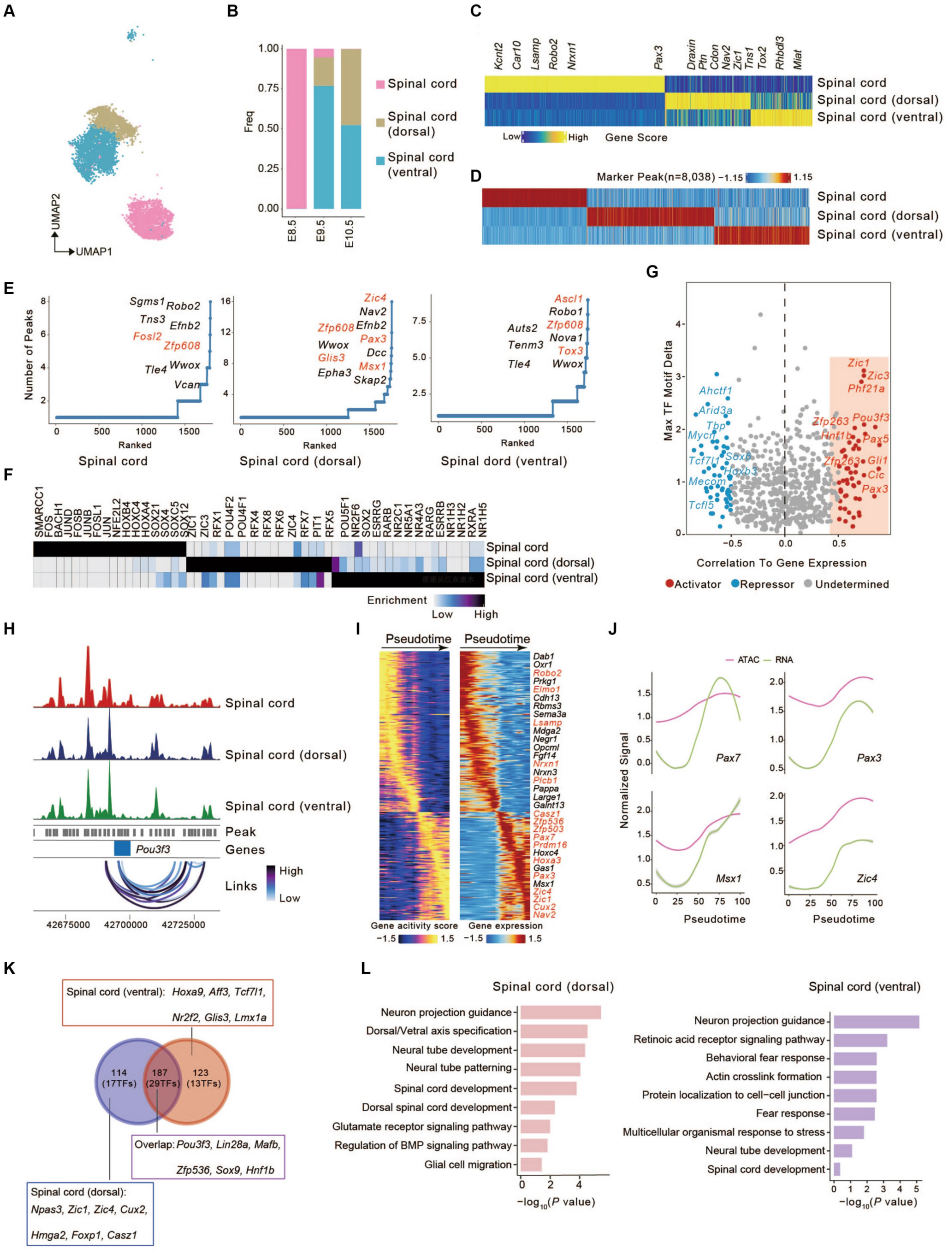
Identification of candidate correlated transcription factors governing spinal cord cell fate. **(A)** UMAP visualization of cells from spinal cord lineages. **(B)** The bar graph shows the proportion of three cell types. Colors match the cell types in **(A)**. **(C)** Heatmap of marker genes across spinal cord lineages calculated from scATAC-seq gene scores. Each column represents a unique marker gene. The color represents the normalized gene score of the marker genes in cell types. **(D)** Heatmap represents the specific marker peak across the spinal cord lineages. Each column represents an individual marker peak. The color represents the normalized marker peak accessibility across three cell types. **(E)** The number of significantly correlated peaks for each gene across three cell types. TFs were colored in red. **(F)** Heatmap of TF motifs enriched in cell-type marker peak. **(G)** Scatterplot showing the correlation between the expression of TFs and their binding motifs in the accessible chromatin region. Putative transcriptional activators and repressors are shown in red and blue, respectively. **(H)** Aggregated scATAC-seq tracks at the genomic regions near *Pou3f3* with peak co-accessibility (Co-access) across three cell types. **(I)** Heatmaps depicting consistent genes of gene score (left) and gene expression (right) for the spinal cord (dorsal) lineages. **(J)** Pseudotime-dependent chromatin accessibility (purple) and gene expression (green) change along the spinal cord (dorsal) lineages. **(K)** Venn plot shows the overlap of genes between **(I)** and (Supplementary Figure S4A). **(L)** Barplots showing the Gene Ontology (GO) of identified pseudotime-dependent genes along the spinal cord (dorsal) and spinal cord (ventral).

Multiple signaling pathways, such as sonic hedgehog (SHH), bone morphogenetic proteins (BMP), and WNT signalings, play a vital role in establishing dorsal-ventral axis of a developing embryo (Rogers and Schier, 2011; Sagner and Briscoe, 2017). The vertebrate neural tube has become a major model for understanding the principles of cell fate determination. Various morphogenic activities emanating from the dorsal and ventral poles of the spinal cord form the dorsal-ventral axis (Le Dréau and Martí, 2012; Lai et al., 2016). To decipher the underlying molecular mechanism of neural tube development, we extracted and further investigated the cells defined as spinal cord, spinal cord (dorsal), and spinal cord (ventral) in our scATAC-seq dataset (Figure 2A), with the spinal cord distributed only at E8.5 and the other two mainly between E9.5 and E10.5 (Figure 2B). After E8.5, spinal cord shift toward spinal cord (dorsal/ventral), displaying a transition from neural progenitor cells to neurons (Figures 2A,B) (Alaynick et al., 2011; Qiu et al., 2022). A total of 850 cell types-specific genes were identified based on gene activity scores across these cell types. The spinal cord was annotated by accessibility near *Kcnt2*, *Car10*, *Lsamp*, *Robo2*, *Nrxn1*, *Plcb1*, and *Pax3*; the spinal cord (dorsal) was annotated by accessibility near *Nav2*, *Draxin*, *Cdon*, *Ptn*, and *Zic1*; and the spinal cord (ventral) was annotated by accessibility near *Tns1*, *Rhbdl3*, *Tox2*, and *Miat* (Figure 2C). Applying the peak calling algorithm MACS2 (Zhang et al., 2008), 8,038 differential peaks in only one or two cell types were identified (Figure 2D).

Mapping differential peaks to the genome enabled us to predict the impact of *cis*-regulatory element (CREs) associated with genes on identifying critical developmental regulators. We tabulated the number of peaks near each gene and ranked them accordingly. Genes having frequent open chromatin domains were deemed higher accessibility and may be more important in cell fate-determining. We found that *Fosl2* (Yin et al., 2023) in the spinal cord, *Zic4* in the spinal cord (dorsal) (Aruga et al., 1996; Ding et al., 2004) and *Ascl1* in spinal cord (ventral) (Kicheva et al., 2014; Misra et al., 2014) have frequent open chromatin domains, which determines the transition of spinal cord progenitor cells to neurons of the dorsal or ventral spinal cord (Figure 2E).

To investigate the transcription factor (TFs) potentially driving the regulatory programs in developing spinal cord, we assayed for TF motif enrichment in differential peaks (Figure 2F). A number of TFs in the same family were deemed enriched due to the similarities in DNA binding motifs. Top enrichment motifs in spinal cord [HOX family (HOXC4, HOXB4, HOXA4)], the members of which establish the distinct neuronal cell fates strongly agree with established spinal cord TFs in the literature (Prince et al., 1998; Sagner and Briscoe, 2019). Similarly, we observed enrichment of ZIC family motifs (ZIC1, ZIC3) and RFX family motifs (RFX4, RFX2, RFX6, RFX3) in spinal cord (dorsal) (Blackshear et al., 2003; Ashique et al., 2009), ZIC1 have also been shown to be important for the early specification of the spinal cord (dorsal) (Aruga et al., 2002). The nuclear receptor subfamily (NR5A1, NR4A3, NR1H4) motifs in spinal cord (ventral) (Figure 2F) (Kon et al., 2015; Ghazale et al., 2019). To investigate the motif activity in spinal cord lineages, we employed chromVAR to measure the deviation z scores of TF motif and then calculated the correlation between deviation z scores of motifs and the TF expression in spinal cord lineages (Schep et al., 2017). We identified 53 putative activators and 50 repressors (Figure 2G), such as *Zic1*, *Pou3f3*, *Zfp263*, and *Pax3*. Additionally, *Pou3f3* gene locus coaccessible peaks were found in all three spinal cord lineages, suggesting potential coaccessible CREs (Figure 2H) (Misra et al., 2014). These chromatin behaviors are significant for cell fate specification in spinal cord lineages.

### Reconstruction of developmental trajectories during spinal cord lineages

To understand the dynamic change in the development and differentiation of the spinal cord in the dorsal or ventral direction, we established trajectories based on sequential differentiation states. We identified several genes with similar dynamic patterns for *cis*-elements and gene expression (Figure 2I and Supplementary Figure S4A). Besides some genes shared between dorsal and ventral development, others showed differential characteristics (*Plcb1*, *Elmo1*, *Lsamp*, *Nrxn1* in dorsal direction; *Aldh1a2*, *Crabp2*, *Prkg1*, *Nrg3* in ventral direction) (Figure 2I and Supplementary Figure S4A). This also reflects the characteristics of different neuron subgroups of the spinal cord (dorsal) lineage (*Casz1*, *Nav2*, and *Prdm16*) and spinal cord (ventral) lineage (*Nes*, *Zfp536*, *Nr2f1*, and *Hoxc5*). In the spinal cord (dorsal) lineage, we observed that *Pax7*, *Pax3*, *Msx1*, and *Zic4* maintained chromatin accessibility at this locus, and gene expression increased along the pseudotimes as accessibility increased (Figure 2J). The same was seen in the spinal cord (ventral) lineage for *Pax7*, *Nr2f1*, *Hoxb9*, and *Hoxc4* (Supplementary Figure S4B).

We compared pseudotime dynamics genes from the dorsal and ventral directions. We found that, apart from some shared genes (187 genes (29 TFs): *Pou3f3*, *Lin28a*, *Sox9*, etc.), there were distinct characteristics for the development towards the dorsal [114 genes (17 TFs): *Npas3*, *Zic1*, *Zic4*, etc.] or ventral direction [123 genes (13 TFs): *Hoxa9*, *Aff3*, *Tcf7l1*, etc.] (Figure 2K). *Zic1*, which regulates key marker genes (*Nr2f1*, *Foxp1*, *Cux2*, and *Hmga2*) with high motif activity, is essential for the early specification of dorsal neurons. *Npas3,* which is mainly expressed in the late stage of spinal cord (dorsal) differentiation (Kamm et al., 2013), was also supported by our data (Figure 2K and Supplementary Figure S4C). We also identified additional TFs that regulate spinal cord (ventral) fate, such as *Hoxc5*, *Glis3*, *Hoxa9*, *Tcf7l1*, and *Nr2f2* (Supplementary Figure S4D). Finally, gene enrichment analysis revealed that pseudotime dynamics genes associated with the dorsal direction were linked to “Neuron projection guidance,” “Neural tube development,” “Glutamate receptor signaling pathway,” and “Regulation of BMP signaling pathway”; those related to the ventral direction were related to “Behavioral fear response,” “Actin crosslink formation” and “Multicellular organismal response to stress” (Figure 2L).

### Dynamic epigenomic landscapes across developmental trajectories in mouse somitogenesis

**Figure 3:**
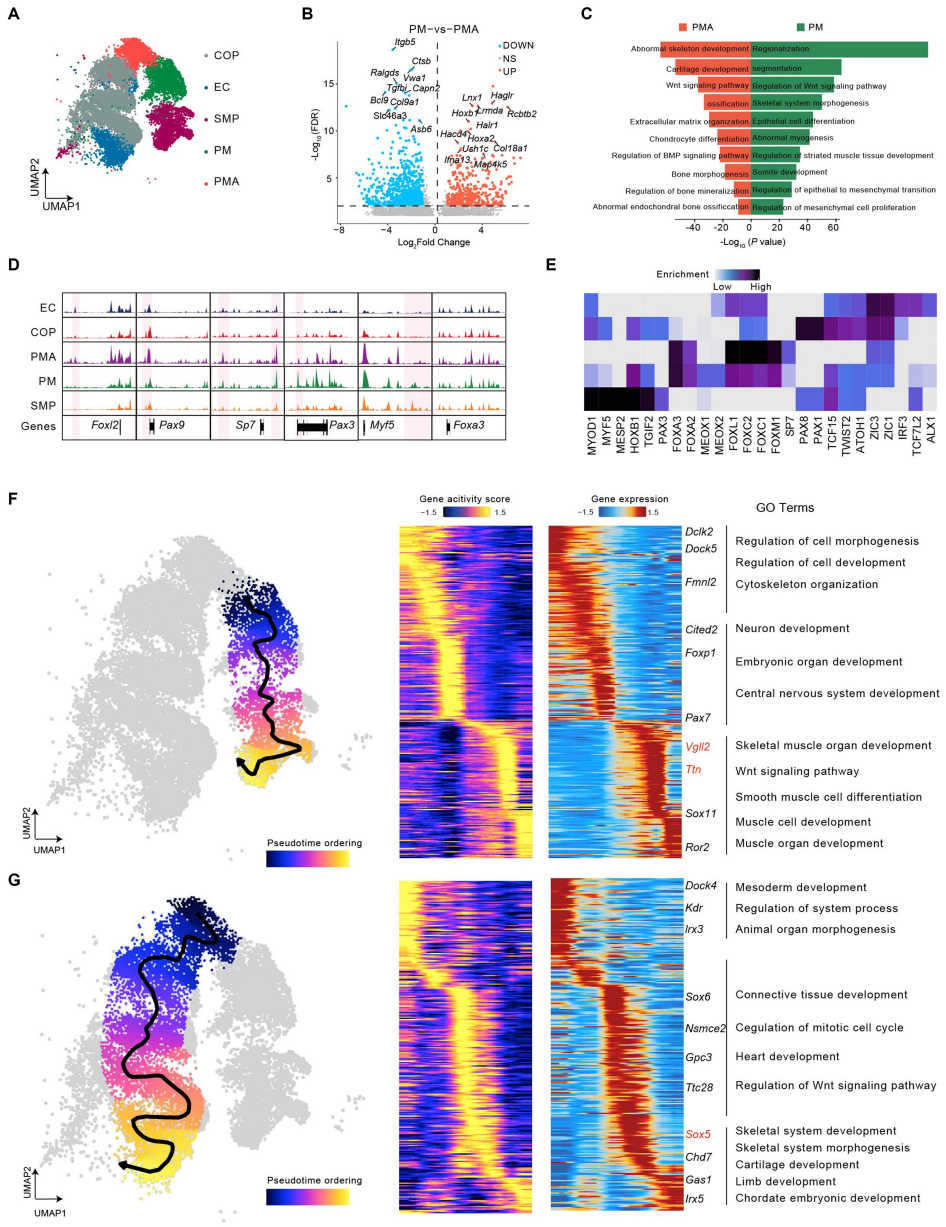
Dynamic epigenomic landscapes across developmental trajectories in mouse somitogenesis. **(A)** UMAP visualization of cells in mouse somitogenesis. **(B)** Volcano plot of differential peaks compared between PM and PMA. **(C)** GREAT Gene ontology (GO) annotation of differential peaks upregulated in PM (green) and in PMA (red). **(D)** Genome-wide coverage tracks from each cluster around the *Foxl2*, *Pax9*, *Sp7*, *Pax1*, *Myf5*, and *Foxa3* gene loci in somitogenesis. Specific peaks activated in different clusters have been marked in pink region. **(E)** The heatmap of enriched motifs from each cluster in somitogenesis. **(F)** The scatter plot covering UMAP demonstrated the differentiation trajectory from PM to skeletal muscle progenitors (left). Heatmaps depicting consistent genes of gene score (middle) and gene expression (right) for PM lineage. **(G)** as **(F)** but with respect to PMA lineage. Key genes for lineages have been marked, and GO annotations were indicated.

To investigate the heterogeneity involved in somitogenesis, we performed UMAP analysis of cells in somitogenesis and identified five subclusters, two of which maintained chromatin accessibility near paraxial mesoderm precursor markers were annotated as paraxial mesoderm (PM) and paraxial mesoderm A (PMA) (Figure 3A and Supplementary Figure S2). Differential chromatin accessibility analysis revealed 3,602 and 5,755 peaks specifically open in PM and PMA, respectively. In particular, *Hoxb1*, *Map4k5*, and so on had higher accessibility in the promoter region for PM, while *Ctsb* and *Asb6* and so on had higher accessibility for PMA (Figure 3B). Gene Ontology (GO) analysis using Genomic Regions Enrichment of Annotations Tool (GREAT) (Mclean et al., 2010) showed that upregulated peaks in PM were associated with regionalization, somite development and skeletal muscle system development, while those in PMA were mostly associated with ossification (Figure 3C). These results implied that the PM was involved in skeletal muscle differentiation and PMA potentially develop into the skeleton.

We then highlighted subcluster-specific peaks using coverage track analysis, indicating that *Sp7* and *Pax3* showed chromatin accessibility specifically in PMA and PM. According to previous studies, *Sp7* (*Osterix*) knockout induces an abnormal bone morphogenesis phenotype and immature osteocytes in mice (Nakashima et al., 2002; Zhou et al., 2010) and is also associated with human skeletal diseases (Sinha and Zhou, 2013). *Pax3*, a member of the PAX family, is a major regulator of myogenesis and skeletal muscle development (Buckingham and Relaix, 2007; Buckingham and Relaix, 2015; Boudjadi et al., 2018). In addition, *Foxl2*, *Pax9*, and *Myf5* showed specific chromatin accessibility in early chondrocytes (EC), COP, as well as skeletal muscle progenitors (SMP), respectively. Previous studies have found that *Foxl2* can modulate cartilage development (Marongiu et al., 2015), *Pax9* may be vital for chondrocyte growth (Peters et al., 1999), and *Myf5* is an important regulator of skeletal muscle system development (Zammit, 2017). In contrast, *Foxa3* maintained relatively high accessibility in each subcluster (Figure 3D), which suggested a crucial role for both skeletal and skeletal muscle development. Consistent with our data, a previous study revealed the essential role of *Foxa3* in chondrocyte differentiation in mice (Ionescu et al., 2012). Motif enrichment analysis across all subclusters showed a series of subcluster-specific motifs, such as *Myf5,* mainly enriched in skeletal muscle progenitors. However, some motifs are enriched in several subclusters. For instance, *Zic1* and *Zic3* were enriched in all subclusters except skeletal muscle progenitors (Figure 3E). Finally, we constructed the trajectory from PM into skeletal muscle progenitors and found a series of genes with pseudotime dynamic patterns (Figure 3F). By integrating scATAC-seq and scRNA-seq, we observed a group of genes like *Vgll2* and *Ttn* gradually increased expression with increased chromatin accessibility along the pseudotime. GO term enrichment analysis shown those genes were mainly enriched in muscle-related processes, including muscle cell development, skeletal muscle organ development, Wnt signaling pathway, and so on (Figure 3F). The pseudotime analysis for PMA to chondrocyte and osteoblast progenitors and then to early chondrocytes showing the pseudotime dynamic genes such as *lrx3*, *Sox6*, *Sox5* (Tani et al., 2020), *Chd7* and *Irx5* were mainly enriched in the skeletal developmental process, including skeletal system development, cartilage development and chordate embryonic development (Figure 3G). Overall, our findings showed the heterogeneity and epigenetic dynamics during somitogenesis.

### Predicting cell types associated traits and diseases in embryo development

The vast majority of SNPs localized to noncoding regions of the genome and operate in a cell-type-specific manner (Prescott et al., 2015; Nott et al., 2019; Krausgruber et al., 2020). Given the degree of conservation of chromatin accessibility between mice and humans, the genomic location in human was matched to the mouse orthologue (Vierstra et al., 2014; Moore et al., 2020). Mouse scATAC-seq data help to understand the cell-type-specific effects of genetic variation underlying complex human traits (Cusanovich et al., 2018; Li et al., 2021; Miao et al., 2021).

**Figure 4:**
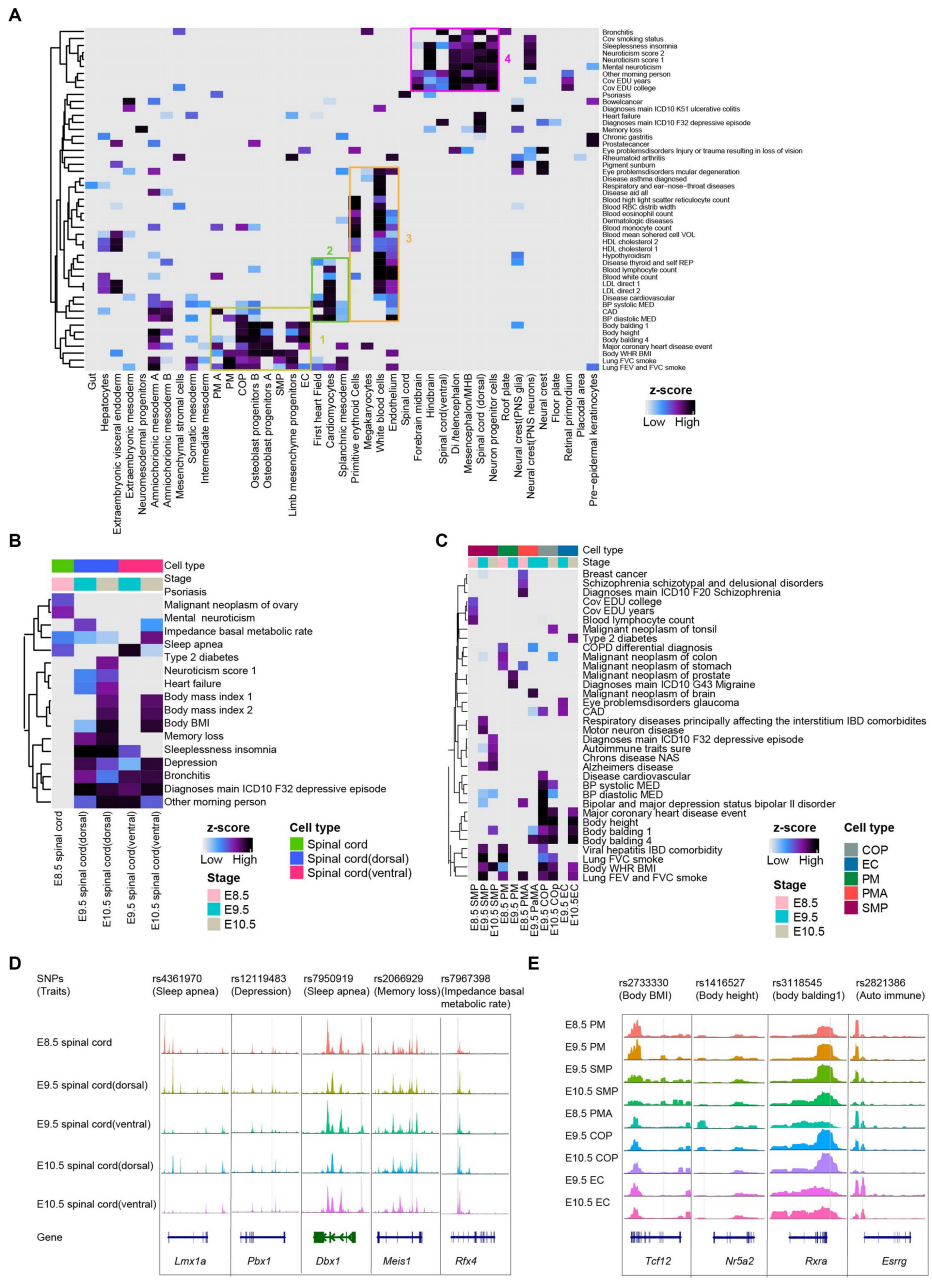
Mouse embryo chromatin accessibility profiles associated with human traits and genetic diseases. **(A)** Heatmaps depicting enrichment for traits across all cell types with scATAC-seq dataset. The horizontal axis is the cell types from global clustering, and the vertical axis is the traits/diseases in the GWAS database. The boxes represented selected significant feature enrichment. **(B)** Heatmaps depicting enrichment for the phenotype in spinal cord lineages from E8.5-E10.5. **(C)** Heatmaps showing enrichment for the phenotype in somitogenesis from E8.5-E10.5. **(D,E)** Examples of the benefits of snATAC-seq for pinpointing cell types whereby candidate spinal cord lineage **(D)** and somitogenesis **(E)** regulatory variants are acting. Genes in the sense and antisense directions are shown in blue and green. The location of each human SNPs is depicted by a vertical gray line.

We applied LDSC analysis (see Methods) to the mouse embryonic scATAC-seq datasets. We linked human SNPs from the UK Biobank^4^ to orthologous coordinates of the mouse chromatin accessibility regions to calculate the enrichment of traits across the chromatin accessibility in each annotated cluster. As a general trend, 49 traits were enriched (coefficient *p* < 0.01) in at least one cell type (Figure 4A and Supplementary Table S1). In addition, we observed enriched heritability for neurological traits (e.g., “sleeplessness” and “neuroticism”) shown in neural clusters (Figure 4B). In line with expectations, somitogenesis-related traits (e.g., “body height” and “body balding”) in clusters correspond to somitogenesis cells (Figure 4C). After lifting over human SNPs to orthologous coordinates in the mouse genome (see Section Methods), several orthologous GWAS locus observed a strong open chromatin region in the spinal cord and somitogenesis (Figures 4D,E). As an example, we identified rs4361970 region at *Lmx1a* locus. This locus has been implicated in sleep apnea and robustly associated with the expression of critical genes involved in mitochondrial functions (Chizhikov and Millen, 2004; Radulovacki et al., 2004; Doucet-Beaupré et al., 2016; Kostin et al., 2021). We observed that rs4361970 was most accessible in E8.5 spinal cord cells (Figure 4D). We also found rs2733330 region at *Tcf12* locus (Cao et al., 2010; Ke et al., 2021; Wang et al., 2022). *Tcf12* bind to the E-box regions of *Myod* and *Myog,* contributing to SMP (Parker et al., 2006). We observed that rs2733330 was most accessible in SMP (Figure 4E).

## Discussion

In this study we have generated a cellular resolution chromatin accessibility map of the developing mouse embryo and identified vital cell type-specific regulatory networks in organogenesis. The difference between E8.5 and the other two time points are significant, whereas the difference between E9.5 and E10.5 are small, suggesting that E8.5 cell type is closer to gastrulation stage cells. By integrating previously published scRNA-seq datasets (Qiu et al., 2022) and spatial RNA-seq (Chen et al., 2022) with our scATAC result, we constructed spatial maps of the epigenetic landscape in mouse organgenesis.

The spinal cord connects and allows communication between the brain and surrounding organs, and its dorsal-ventral axis and neuronal subtypes have been reported (Delile et al., 2019; Shu et al., 2022). However, the epigenetic basis of cell fate decisions related to spinal cord development along the dorsal-ventral axis remains unclear. Here, we defined the cellular differentiation trajectories, characterize regulatory dynamics, and identify key driving TFs for spinal cord development.

Previous studies have mainly focused on somitogenesis, however, the dynamics of epigenetic regulation of PM development in somitogenesis remain largely unknown. We present the first report of the epigenomic regulatory mechanism driving paraxial mesoderm to develop into sclerotome and dermomyotome, which form the axial skeleton, and skeletal muscle progenitors. We identified the differential peaks between PM and PMA to support this hypothesis. Complementing these studies, our results suggested that motifs (such as *Sp7*, *Pax3*, *Foxl2*, *Pax9*, *Myf5*) are involved in the differentiation and development of the skeleton and skeletal muscle. Furthermore, the differentiation trajectories indicated a pseudotime from PM to skeletal muscle progenitors, involving the Wnt signaling pathway, which is vital for the skeletal muscle development (Girardi and Le Grand, 2018). Additionally, our results showed that potential differentiation from PMA to early chondrocytes, and skeleton development-related signaling contribute to this process. In conclusion, our findings depicted the dynamics of epigenetic regulation for the differentiation of mouse paraxial mesoderm to the skeleton and skeletal muscle at single-cell resolution.

GWAS signals have been successful in identifying nucleotide variations with specific traits/diseases (Maurano et al., 2012). SNPs associated with human traits/diseases are located in regions with murine cell type-specific and developmental stage-specific regulatory activity, providing potential targets for subsequent pathogenesis studies and treatments.

## Data availability statement

The datasets presented in this study can be found in online repositories. The names of the repository/repositories and accession number(s) can be found at: https://db.cngb.org/search/project/CNP0003941/.

## Ethics statement

The animal study was reviewed and approved by the institutional review board of BGI ethical clearance.

## Author contributions

QD, SW, ChuanL, and LL conceived the idea. GL collected the samples and generated the data. JX, YY, XL, MC, SD, and XC assisted with the experiments. SW analyzed the data with the assistance of QL, WF, SH, HZ, and WM. QD wrote the manuscript with the input of SW, YL, ChangL, and LL supervised the study. ChuanL and YL provided helpful comments on this study and revised the manuscript. All authors contributed to the article and approved the submitted version.

## Funding

This study were supported by the Guangdong Basic and Applied Basic Research Foundation (2021B1515120075) and Shenzhen Bay Laboratory (SZBL2019062801012).

## Conflict of interest

The authors declare that the research was conducted in the absence of any commercial or financial relationships that could be construed as a potential conflict of interest.

## Publisher’s note

All claims expressed in this article are solely those of the authors and do not necessarily represent those of their affiliated organizations, or those of the publisher, the editors and the reviewers. Any product that may be evaluated in this article, or claim that may be made by its manufacturer, is not guaranteed or endorsed by the publisher.

## Footnotes

^1^http://github.com/shiquan/PISA

^2^https://broadinstitute.github.io/picard

^3^https://github.com/bulik/ldsc/wiki/Cell-type-specific-analyses

^4^https://www.ukbiobank.ac.uk/
