# Supplementary figures and images for: Single-cell chromatin accessibility profiling of cell-state-specific gene regulatory programs during mouse organogenesis
Source: Front Neurosci. 2023 Jun 27;17:1170355. doi: 10.3389/fnins.2023.1170355 (PMC10333525; doi:10.3389/fnins.2023.1170355)

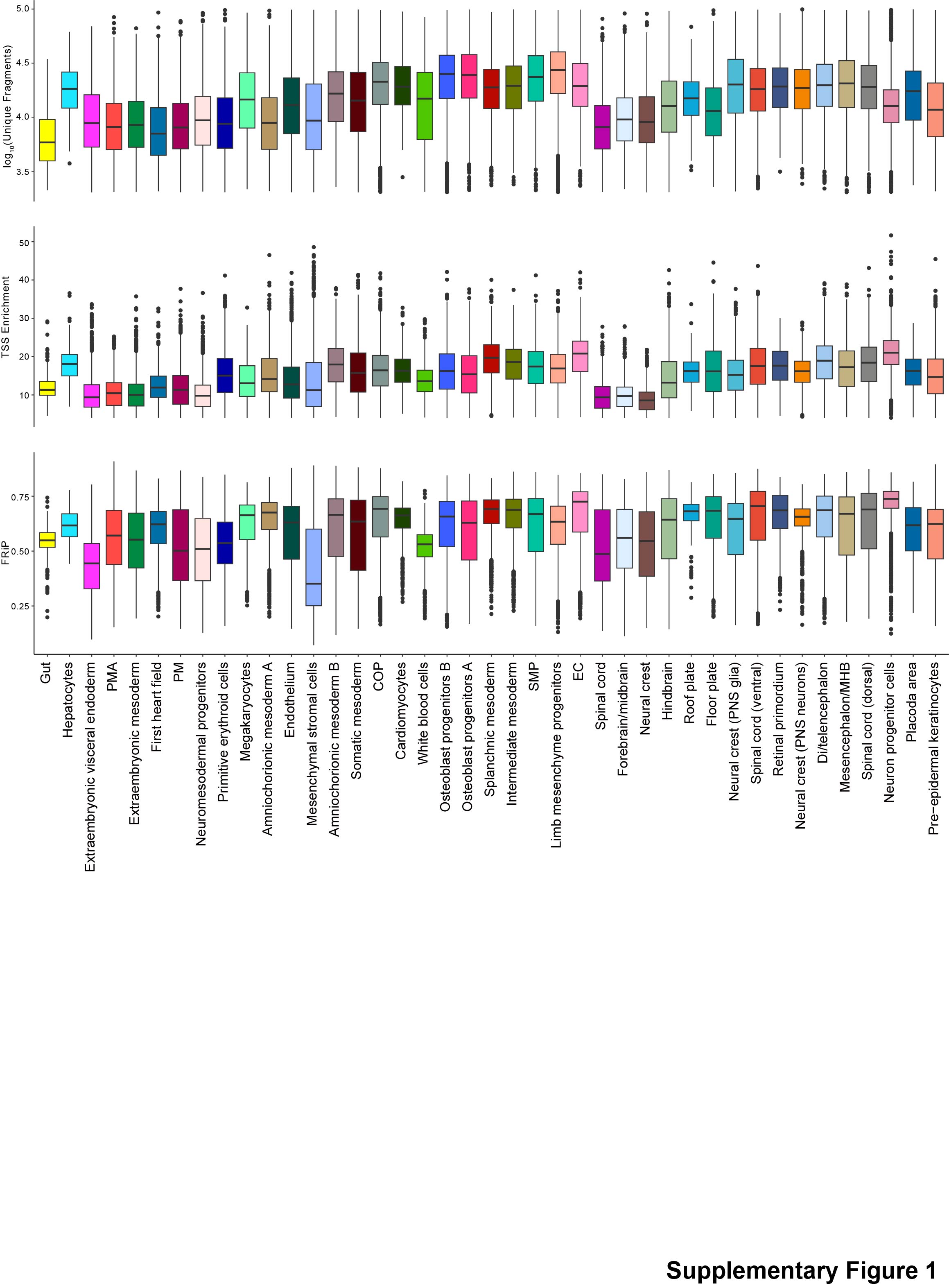

Supplement: Supplementary file 1 [file Image_1.JPEG]

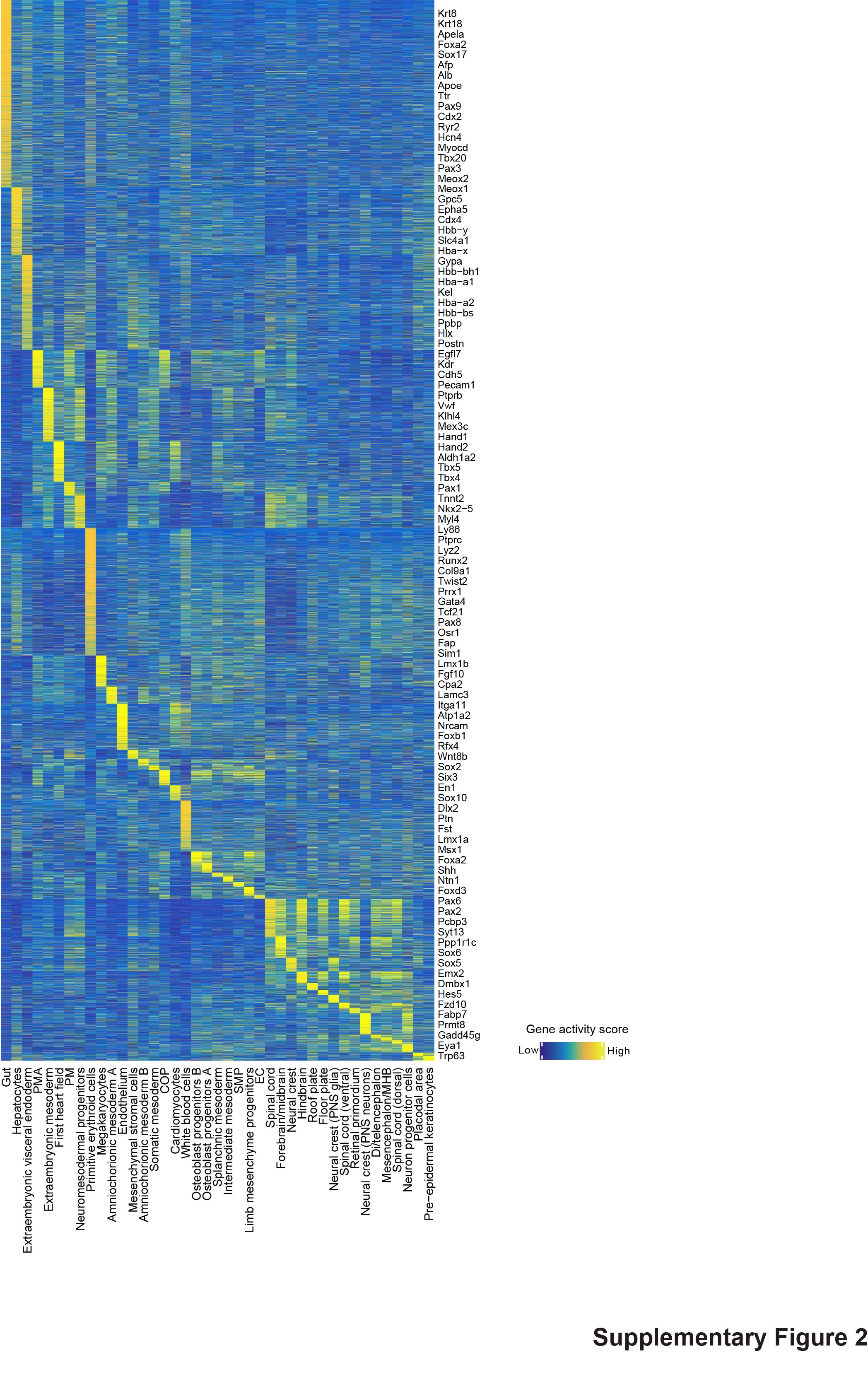

Supplement: Supplementary file 2 [file Image_2.JPEG]

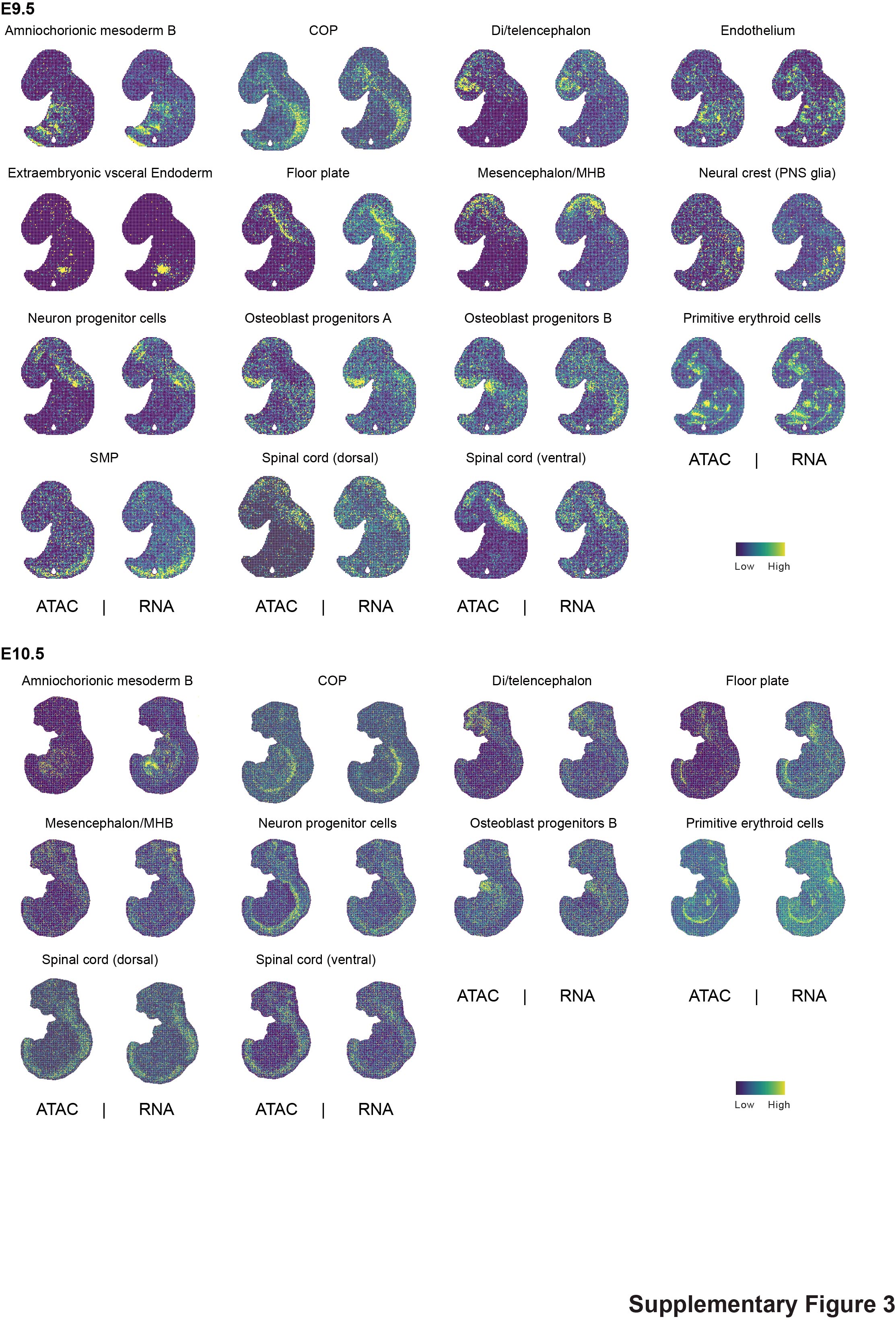

Supplement: Supplementary file 3 [file Image_3.JPEG]

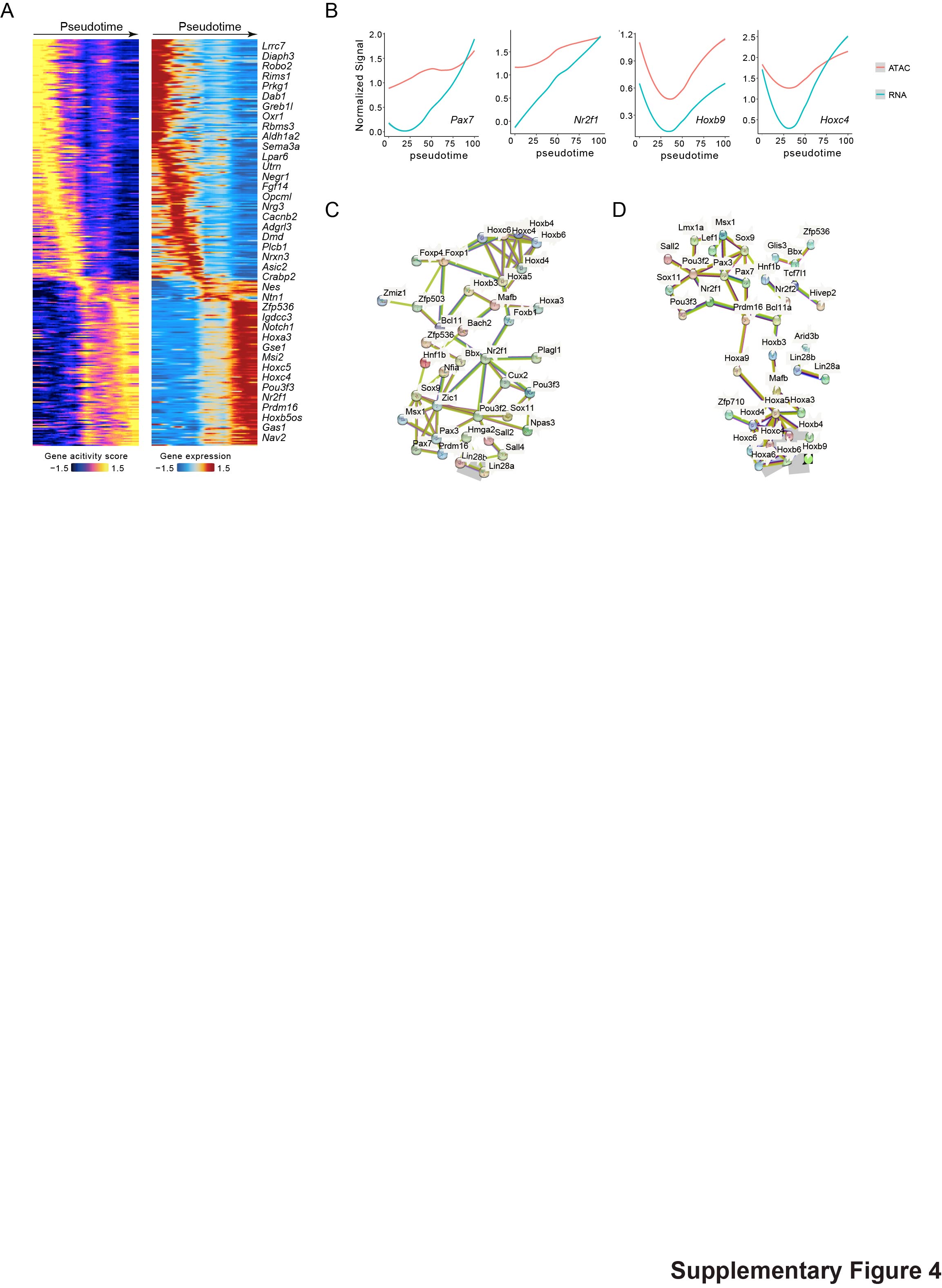

Supplement: Supplementary file 4 [file Image_4.JPEG]
